# An Autonomous Path Planning Model for Unmanned Ships Based on Deep Reinforcement Learning

**DOI:** 10.3390/s20020426

**Published:** 2020-01-11

**Authors:** Siyu Guo, Xiuguo Zhang, Yisong Zheng, Yiquan Du

**Affiliations:** School of Information Science and Technology, Dalian Maritime University, Dalian 116026, China; guosy@dlmu.edu.cn (S.G.); zhengyisong@dlmu.edu.cn (Y.Z.); duyiquan@dlmu.edu.cn (Y.D.)

**Keywords:** unmanned ships, deep reinforcement learning, DDPG, autonomous path planning, end-to-end, collision avoidance

## Abstract

Deep reinforcement learning (DRL) has excellent performance in continuous control problems and it is widely used in path planning and other fields. An autonomous path planning model based on DRL is proposed to realize the intelligent path planning of unmanned ships in the unknown environment. The model utilizes the deep deterministic policy gradient (DDPG) algorithm, through the continuous interaction with the environment and the use of historical experience data; the agent learns the optimal action strategy in a simulation environment. The navigation rules and the ship’s encounter situation are transformed into a navigation restricted area, so as to achieve the purpose of planned path safety in order to ensure the validity and accuracy of the model. Ship data provided by ship automatic identification system (AIS) are used to train this path planning model. Subsequently, the improved DRL is obtained by combining DDPG with the artificial potential field. Finally, the path planning model is integrated into the electronic chart platform for experiments. Through the establishment of comparative experiments, the results show that the improved model can achieve autonomous path planning, and it has good convergence speed and stability.

## 1. Introduction

With the density of maritime traffic increasing, various types of marine accidents frequently occur. 80% of marine accidents are caused by human factors, according to the world maritime disaster records that were filed by international maritime organization (IMO) from 1978 to 2008 [1]. Therefore, improving the autonomous driving level of ships has become an urgent problem to be solved. On the other hand, the environment that is faced by ships is more and more complex. In some cases, it is not suitable for manned ships to go to the workplace to carry out tasks, while unmanned ships are more suitable for dealing with the complex and changeable harsh environment at sea. This requires unmanned ships to have the ability of autonomous path planning and obstacle avoidance, so as to efficiently complete tasks and enhance the comprehensive operation ability [2].

With their strong autonomy and adaptability, unmanned ships have gradually become the new research direction pursued by the current industry [3]. Unmanned ships can not only perform tasks independently in dangerous sea areas, but also cooperate with manned ships to improve work efficiency. They are widely used in marine exploration, military tasks, material transportation, and other fields. Autonomous path planning is an essential key technology in order to improve the self-determination ability of unmanned ships. The autonomous path planning of unmanned ships needs to optimize the path in the safe navigation area according to certain navigation rules and crew experience. It can safely avoid obstacles and independently plan an optimal trajectory from the known starting point to the target point. Unmanned ships often face complex and variable navigational environments. Therefore, it is necessary to adopt a continuous and effective method for controlling the trajectory of the ship during the voyage, thus ensuring the safety of the ship [4]. The research directions of unmanned ships are mainly divided into autonomous path planning, navigation control, autonomous collision avoidance, and semi-autonomous task execution. Autonomous path planning plays a key role in ship automation and practical application, as the basis and premise of autonomous navigation [5]. In the actual navigation process, ships often meet with other ships, which requires reasonable methods to guide ships to avoid other ships and achieve the target point. Therefore, it is valuable to consider how to avoid dynamic obstacles in the process of path planning. The unmanned ship path planning method can guide the ship to take the optimal action and avoid other obstacles. At the same time, it can also be divided into the navigable area and the obstacle area by the method according to the obstacle information to realize the function of avoiding the obstacle in the local area. Many scholars have completed relevant research and experiments to solve the problem of autonomous path planning for unmanned ships. However, traditional path planning methods usually require relatively complete environmental information as prior knowledge, and it is quite difficult to obtain surrounding environment information in an unknown sea environment. Moreover, the traditional algorithm has a large amount of computation, which makes it difficult to realize the real-time behavior decision-making of ships, resulting in large mistake information of path planning.

In recent years, artificial intelligence technology has been rapidly developed and applied. Deep Learning (DL) [6] and Reinforcement Learning (RL) [7] have achieved great success in many fields. DL has a strong perceptual ability and RL has decision-making ability. DRL [8] is obtained by combining the advantages of DL and RL, which provides a solution to the perceptual decision-making problem of complex systems. DRL can effectively solve the problem of continuous state space and action space. It directly takes original data as input and output results as execution action, realizes an end-to-end learning mode, and greatly improves the efficiency and convergence of the algorithm. At present, DRL has been widely used in robotic control [9], automatic driving [10,11,12], financial prediction [13], traffic control [14], and other fields. Artificial intelligence technology is gradually infiltrating into various fields and the concept of unmanned autonomy is getting closer. As an important part of the transportation field, unmanned ships are moving forward in the direction of intelligence and autonomy.

RL has attracted widespread attention in recent years, emphasizing the learning of agents from the environment to behavioral mapping and seeking the most accurate or optimal action decisions by maximizing the value function. Mnih, V et al. [15] proposed a Deep Q-Network (DQN) algorithm, which opened a new era of DRL. The DQN algorithm utilizes the powerful function fitting ability of the deep neural network to avoid the huge storage space of the Q table, and the experience replay memory and target network are used to enhance the stability of the training process. At the same time, DQN implements an end-to-end learning method, and only original data are used as input, and the output result is the Q value of each action. The DQN algorithm has achieved great success in discrete action, but it is difficult to achieve high-dimensional continuous action. If continuously changing action is infinitely split, the number of actions exponentially increases with increasing degrees of freedom, which leads to the problem of latitude catastrophe and can cause great training difficulties. In addition, simply discretizing the action removes important information regarding the structure of the action domain. The Actor-Critic (AC) algorithm [16] is capable of handling continuous action problems and it is widely used in continuous action spaces. The AC algorithm network structure includes Actor network and Critic network. The Actor network is responsible for outputting the probability value of the action. The Critic network evaluates the output action. In this way, the network parameters are continuously optimized and the optimal action strategy is obtained, but the random strategy of the AC algorithm makes the network difficult to converge. Lillicrap, T.P et al. [17] offered the Deep Deterministic Policy Gradient (DDPG) algorithm to solve the problem of DRL in continuous state action space. DDPG is a model-free algorithm that combines the advantages of the DQN algorithm with an experience replay memory and a target network. At the same time, the AC algorithm that is based on the Deterministic Policy Gradient (DPG) is used to make the network output result a certain action value, which ensures that DDPG can be applied to the continuous action space field. The DDPG can be easily applied to complex problems and larger network structures with simplicity and convergence. Zhu, M et al. [11] proposed a framework for human-like autonomous car-following planning based on DDPG. In this framework, unmanned cars learn from the environment through trial and error. Finally, the path planning model of unmanned cars was obtained and it has good experimental results. This research shows that DDPG can gain insight into driver behavior and can help to develop human-like autopilot algorithms and traffic flow models.

This paper proposes an autonomous path planning model for unmanned ships that are based on the DRL method. The essence of the model is that the agent independently finds the most efficient path through the enumerated method, which might be closer to human manipulation. At the same time, the ship motion is transformed into a continuous motion control problem, so that it conforms to the actual ship’s motion characteristics. The above ideas have been implemented in this paper. Firstly, the interaction mode between ships and environment is analyzed, and a virtual computing environment for unmanned ships autonomous path planning close to the real world is established. Secondly, the model is defined and the network structure and parameters of the DDPG algorithm are set, and the action exploration strategy and reward function are designed. At the same time, the international maritime collision avoidance rules (COLREGS) and crew’s experience are quantified, and the attraction strategy to the target point is added, to ensure the standardization of navigation and avoid the algorithm falling into local optimum. Finally, historical experience data is stored in the memory pool and the neural network parameters are updated by random extraction, thereby reducing the relevance of the data and improving the learning efficiency of the algorithm. Besides, this paper combines the artificial potential field (APF) with the DDPG to obtain the APF-DDPG based autonomous ship autonomous path planning model. The APF-DDPG model has higher decision making power and faster convergence. The model is integrated with the electronic chart platform in order to evaluate the validity and accuracy of the model, and experiments are carried out under the conditions of a single ship encounter and multi-ship encounter, respectively. Moreover, five sets of verification experiments of the DQN, AC, DDPG, APF-DDPG, and Q-learning [18] algorithms are designed as comparative cases. The results show that the APF-DDPG has high convergence speed and planning efficiency, the planned path is more in line with navigation rules, and the autonomous path planning of unmanned ships is realized.

The rest of this paper is structured, as follows. Section 2 reviews the related works. Section 3 presents the autonomous path planning model for unmanned ships that are based on DRL. Section 4 presents a comparative analysis of the simulation experiment process and experimental results. Finally, Section 5 concludes the paper.

## 2. Related Research

At present, research on autonomous path planning for unmanned ships has been carried out at domestic and foreign. The methods include traditional algorithms, such as APF, velocity obstacle method, and A* algorithm, as well as some intelligent algorithms, such as ant colony optimization algorithm, genetic algorithm, neural network algorithm, and other related algorithms of DRL.

In terms of traditional algorithms, Petres, C et al. [19] used APF to construct a virtual gravitational field to guide the autonomous surface vehicles (ASV) to the target point. The ASV avoids obstacles and performs path planning in a complex navigation environment by transforming the navigation restricted area into a virtual obstacle area. The APF has the advantages of high computational efficiency and simple algorithm, but it is necessary to set reasonable potential field parameters to avoid falling into local minimum. The practicality and effectiveness of the algorithm can be guaranteed in the process of unmanned ships autonomous path planning, combined with the COLREGS [20]. Kuwata, Y et al. [21] adopted the velocity obstacle method with COLREGS, and presented a path planning method for unmanned surface vehicles (USV) to navigate safely in dynamic, cluttered environments. The experimental results show that USV can achieve better obstacle avoidance and path planning. Campbell, S et al. [22] showed a USV real-time path planning method that was based on improved A* algorithm, which is integrated with decision-making framework and combined with COLREGS. The results show that the method realizes real-time path planning of the USV in complex navigation environment. However, the A* algorithm relies on the design of the grid map, and the size and number of grids will directly affect the calculation speed and accuracy of the algorithm. Xue, Y et al. [23] introduced a path planning method of unmanned ships that are based on APF, and combined with COLREGS. The experimental showed that the method could effectively realize unmanned ships path search and collision avoidance in complex environments. However, this method is difficult to deal with ships autonomous path planning and collision avoidance problems in an unknown restricted navigation environment.

In addition, many intelligent algorithms, such as genetic algorithms, ant colony optimization algorithms, and neural network algorithms, have also been used in the autonomous path planning problem of unmanned ships. For example, Vettor, R et al. [24] used the optimization genetic algorithm to calculate the environmental information as the initial population to obtain the navigation path that satisfies the requirements. Lazarowska, A et al. [25] proposed the ant colony optimization algorithm to transform ships path planning and collision avoidance problems into dynamic optimization problems, with collision risk and range loss as the objective function. The optimal planning path and collision avoidance strategy were obtained based on the motion prediction of dynamic obstacles. Xin, J et al. [26] adopted the improved genetic algorithm to increase the number of offspring by using the multi-domain inversion. The result shows that the algorithm is superior with a desirable balance between the path length and time-cost, and it has a shorter optimal path, a faster convergence speed, and better robustness. Xie, S et al. [27] proposed a predictive collision avoidance method for under-actuated surface ships based on the improved beetle antenna search (BAS) method. A predictive optimization strategy for real-time collision avoidance is established and COLREGS is used as a constraint condition while considering the minimization of safety and economic cost. The simulation experiments verify the effectiveness of the improved BAS method. However, such intelligent algorithms are usually computationally intensive, and they are mainly used in offline global path planning or auxiliary decision making, and they are difficult to be used for real-time ship action decision problems.

In the field of intelligent ships, the application of DRL to the control of unmanned ships has gradually become a new research field. For example, Chen, C et al. [18] introduced a path planning and maneuvering method for unmanned cargo ships that were based on Q-learning. This method can learn the action reward model and it obtains the best action strategy. After enough rounds of training, the ship can find the right path or navigation strategy by itself. Fu, K et al. [28] proposed a ship rotation detection model based on a feature fusion pyramid network and DRL (FFPN-RL). The ship’s independent guidance and docking function is realized by applying the Dueling Q network to the inclined ship detection task. Yang, J et al. [29] showed autonomous navigation control that is based on relative value iterative gradient (RVIG) algorithm for unmanned ships, and designed the navigation environment of the ship while using Unity3D game engine software. The simulation results showed that the unmanned ships could successfully avoid obstacles and reached the destination in a complex environment. Shen, H.Q et al. [30] offered a method that was based on the Dueling DQN algorithm for automatic collision avoidance of multiple ships, and combined ship manoeuvrability, crew’s experience, and COLREGS to verify the path planning and collision avoidance capability of unmanned ships. Zhang, R.B et al. [31], based on the Sarsa on-policy algorithm, proposed a behavior-based USV local path planning and obstacle avoidance method, and tested in real marine environment. WANG, Y et al. [32] introduced a USV course tracking control plan by combining DDPG algorithm and achieved good experimental results. Zhang, X et al. [33] proposed an adaptive navigation method that was based on maritime autonomous surface ships for hierarchical DRL. The method is combined with the ship maneuverability and navigation rules COLREGS for training and learning. The results show that the method can effectively improve the navigation safety and avoid collision. Zhao, L et al. [34] used the proximal policy optimization (PPO) algorithm, combined with the ship motion model and navigation rules, the unmanned ship autonomous collision avoidance model in the multi-ship environment is proposed. The experimental results show that the model could obtain the time efficiency and collision-free path of multiple ships, and it has good adaptability to unknown complex environments. In addition, as the human factor is composed of navigators’ subjectivity and indeterminacy of the navigation situation in the actual navigation process, it is usually has a game character in the actual ship control process [35]. The DRL overcomes the shortcomings of usual intelligent algorithm, which requires a certain number of samples. At the same time, it has less error and response time.

Many critical autonomous path planning methods have been proposed in the field of unmanned ships. However, these methods are mainly focused on the research of small and medium-sized USV, while the research on unmanned ships is relatively rare, and few experts currently apply the DDPG to unmanned ships path planning. This paper chooses DDPG for unmanned ships path planning, because it has powerful deep neural network function fitting ability and better generalized learning ability. The fitting ability can achieve higher precision when approaching the motion of the ship, and the learning ability can enable the ship to obtain the action output close to the human characteristic from the decision behavior, which is helpful in further developing the human-like traffic models. In addition, the outputs of algorithms, such as Q-learning and DQN, are discrete behaviors, which will lead to the discontinuity and incompleteness of the actions. The DDPG has the advantages of fast convergence speed and continuous action space. When compared with the traditional path planning, the method proposed in this paper has more continuous action output and less decision error when the unmanned ship is sailing.

## 3. Construction of Autonomous Path Planning Model for Unmanned Ships Based on DRL

### 3.1. Deep Reinforcement Learning

As an important field of machine learning, DL can extract high-precision feature samples from the original input data. This method has greatly promoted the development of visual object recognition, object detection, and voice recognition. DL uses deep neural networks (DNN) to automatically learn the features of high-dimensional data. The core idea of DL is to update the network parameters through the back propagation method and then discover the distributed characteristics of the data from the training process.

**Figure 1:**
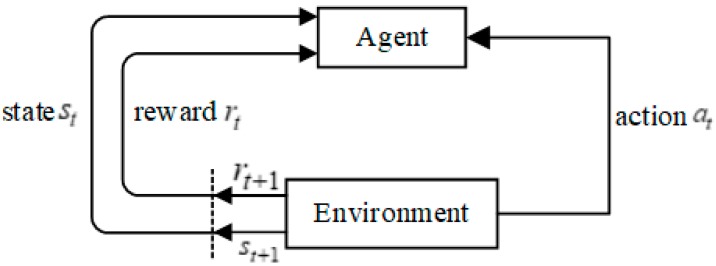
The Principle of Reinforcement Learning.

RL, as another field of machine learning, is widely used in robot control, game simulation, and scheduling optimization. The purpose of RL is to maximize the cumulative reward that was obtained by agents in the training process and learn the optimal strategy to achieve the goal. At the same time, the agent directly interacts with the environment information to replace the large amount of training data by evaluating the action value. The principle is shown in Figure 1. The agent observes the state $s_{t}$ from the environment and makes action $a_{t}$ that is based on a certain policy. The environment then makes feedback reward $r_{t}$ for the executed action and it moves to the new state $s_{t+1}$. The agent uses the new state and reward $r_{t+1}$ to select the action again and keep repeating to maximize the long-term accumulation index of reward.

The DRL is an end-to-end learning method that combines DL and RL, while using the perception ability of DL and the decision-making ability of RL [36]. It has made great progress in continuous motion control and it can effectively solve the shortcomings of traditional unmanned ships in path planning. DDPG is a type of algorithm in DRL that can be used to solve continuous action spaces problems. Among them, deep refers to the deep network structure and policy gradient is the strategy gradient algorithm, which can randomly select actions in the continuous action space according to the learned strategies (action distribution). The purpose of deterministic is to help the policy gradient avoid random selection and to output a specific action value. The DDPG is being gradually applied to the transportation sector, especially in the areas of driverless cars and unmanned ships. These vehicles (buses, small cars, boats, and cargo ships, etc.) often require continuous motion control as well as traffic rules and human operating experience to ensure driving safety. The DDPG can solve the above problems well with its powerful self-learning ability and function fitting ability. Therefore, DDPG has broad prospects and expansion space in the maritime domain.

### 3.2. The Principle of DDPG Algorithm

#### 3.2.1. AC Algorithm

**Figure 2:**
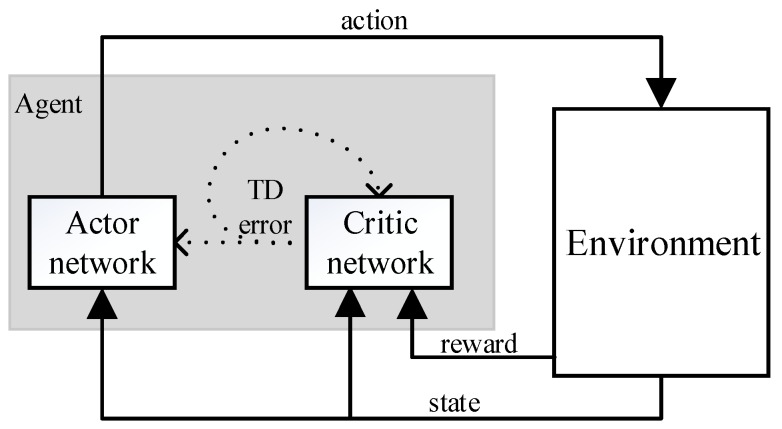
Actor-Critic (AC) algorithm structure.

DDPG is based on AC algorithm, whose structure is shown in Figure 2.

The network structure of the AC framework includes a policy network and an evaluation network. The policy network is called an Actor network, and the evaluation network is called a Critic network. The Actor network is used to select actions corresponding to the DDPG, and the Critic network evaluates the merits and demerits of the selected actions by calculating the value function.

The Actor network and the Critic network are two separate networks that share state information. The Actor network uses state information to generate actions, while the environment feeds back the resulting actions and outputs reward. The Critic network uses state and reward to estimate the value of the current action and constantly adjust its own value function. Meanwhile, the Actor network updates its action strategy in the direction of improving the value of the action. In this cycle, the Critic network evaluates the action strategy by means of a value function, giving the Actor network a better gradient estimate, and finally obtaining the optimal action strategy. It is very important to evaluate the action strategy in the Critic network, which is more conducive to the convergence and stability of the current Actor network. The above features ensure that the AC algorithm can obtain the optimal action strategy with the gradient estimation at a lower variance.

#### 3.2.2. DDPG Algorithm Structure

**Figure 3:**
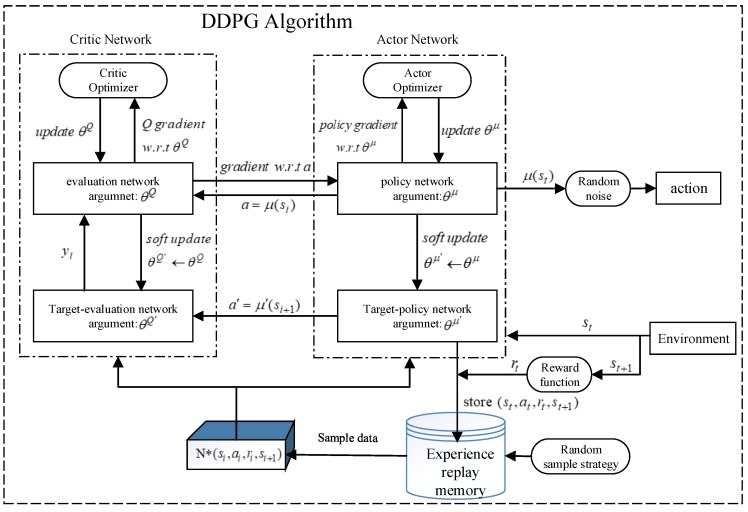
Deep Deterministic Policy Gradient (DDPG) algorithm structure.

Figure 3 shows the structure of DDPG algorithm.

The DDPG algorithm takes the information of the initial state as the input, and the output result is the action strategy $\mu (s_{t})$ calculated by the algorithm. Afterwards, the random noise is added to the action strategy to obtain the final output action, and this process is a typical end-to-end learning mode. When starting the task, the agent outputs an action according to the current state $s_{t}$. The reward function is designed and the action is evaluated in order to verify the validity of the output action, thereby obtaining a feedback reward $r_{t}$ of the environment. The action that is beneficial to the agent to achieve the goal gives a positive reward and, on the contrary, gives a negative reward. Afterwards, the current state information, the action, the reward, and the state information of the next time $\left(s_{t},a_{t},r_{t},s_{t+1}\right)$ are stored in the experience buffer pool. At the same time, the neural network trains experience and continuously adjusts action strategy by randomly extracting sample data from the experience buffer pool, and uses the gradient descent approach to update and iterate network parameters, so as to further enhance the stability and accuracy of the algorithm.

**Figure 4:**
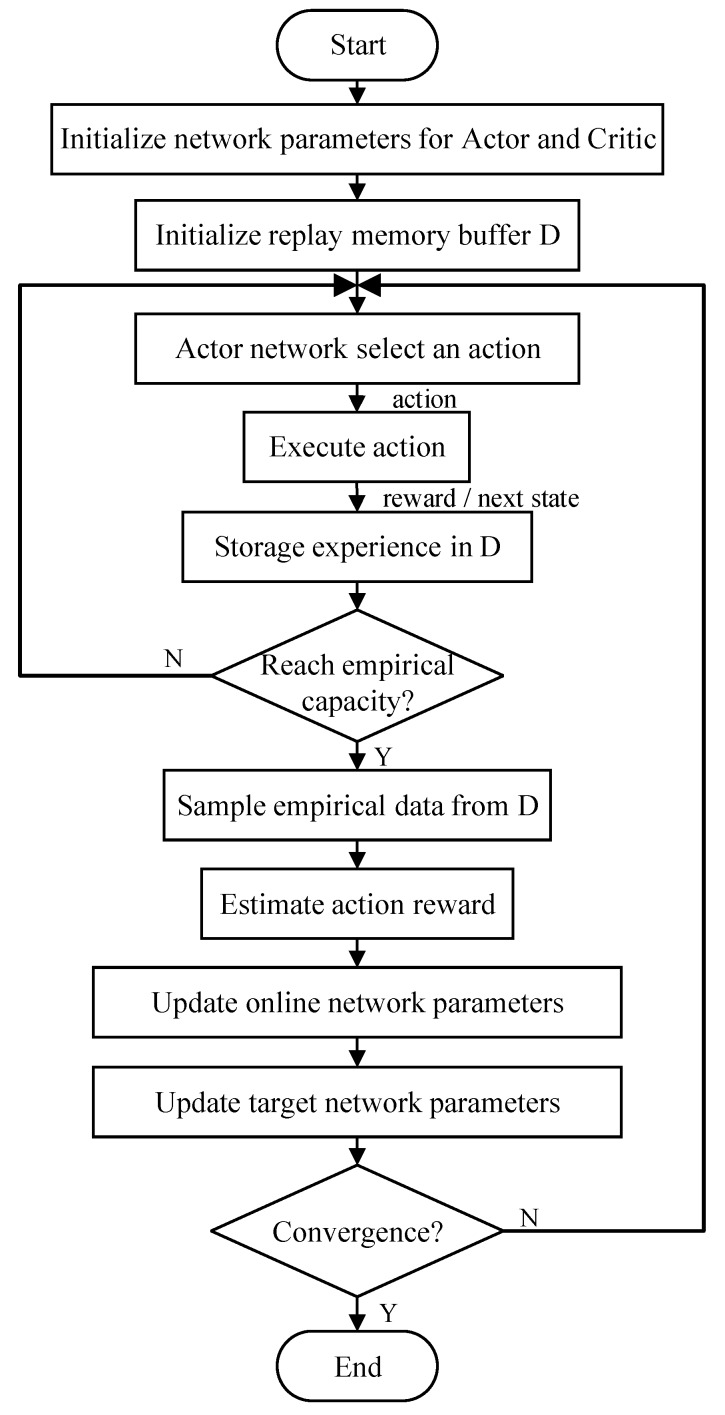
DDPG algorithm flow chart.

DDPG combines with DQN on the premise of AC algorithm in order to further enhance the stability and effectiveness of network training, which makes it more conducive in the filed of solving the problem of continuous state and action space. Additionally, DDPG uses DQN’s the experience replay memory and the target network to solve the problem of non-convergence when using neural network to approximate the function value. Meanwhile, DDPG subdivides the network structure into online network and target network. The online network is used to output the actions in real time, evaluate actions, and update network parameters through online training, which includes online Actor network and online Critic network, respectively. The target network includes target Actor network and target Critic network, which are used to update the value network system and the Actor network system, but not carry out online training and updating of network parameters. The target network and the online network have the same neural network structure and initialization parameters. In the training process, the parameters of the target Actor network and target Critic network are updated in a way of slow change (Soft Replace), instead of directly copying the parameters of online Actor network and online Critic network, so as to further enhance the stability of the training process. The following formulas describe how to update the parameters.

(1) $$\left\{ \begin{array}{l} \theta^{Q^{\prime }}=\tau \theta^{Q}+(1-\tau )\theta^{Q^{\prime }}\\ \theta^{\mu^{\prime }}=\tau \theta^{\mu }+(1-\tau )\theta^{\mu^{\prime }} \end{array}\right.$$

where, $\theta^{\mu }$ and $\theta^{Q}$ are the parameters of the online Actor network and the online Critic network, $\theta^{\mu^{\prime }}$ and $\theta^{Q^{\prime }}$ are the parameters of the target Actor network and the target Critic network, τ≪1 is the approximation coefficient. Figure 4 is the flow chart of the DDPG algorithm.

### 3.3. Structure Design of Autonomous Path Planning Model for Unmanned Ships Based on DDPG Algorithm

The DDPG algorithm is a combination of deep learning and reinforcement learning. Based on this algorithm, this paper designs an autonomous path planning model for unmanned ships. The algorithm structure mainly includes AC algorithm, experience replay mechanism, and neural network. The output action of the model is gradually accurate by using the AC algorithm to output and judge the ship’s action strategy. By using the experience replay mechanism, the historical information of the DDPG algorithm executed in the experimental environment is remembered, and the data are randomly extracted for training and learning. In the course of the experiment, the number of environmental states and behavioral states of the ship is large, so it is necessary to use neural networks for fitting and generalization. The current state of the unmanned ship obtained in the environment is used as the input of the neural network, and the output is the Q value of actions that the ship can perform in the current state. The model can learn the optimal action strategy in the current state by continuously training and adjusting neural network parameters.

#### 3.3.1. Model Structure

**Figure 5:**
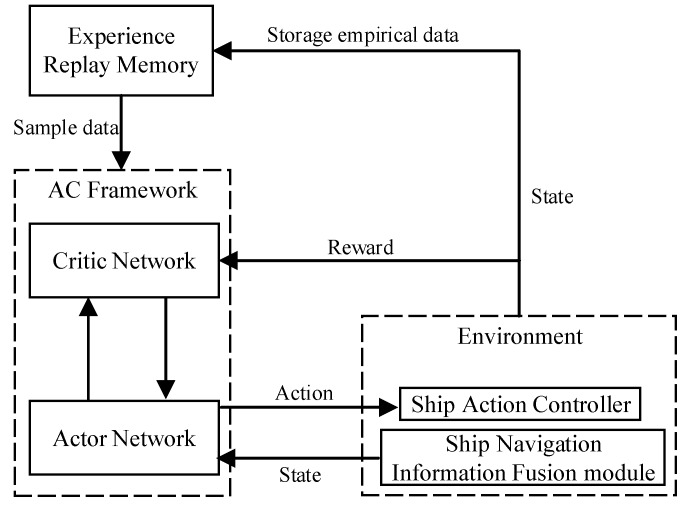
Structure of DDPG algorithm based unmanned ships path planning model.

Figure 5 shows the model structure of unmanned ships path planning based on the DDPG algorithm. The model mainly includes AC algorithm, Environment (Ship Action Controller and Ship Navigation Information Fusion Module), and Experience Replay Memory. Among them, the model obtains the environmental information and the ship’s state data through the Ship Navigation Information Fusion Module, and it serves as the input state of the AC algorithm. The optimal ship action strategy is output, which satisfies the ship’s maximum cumulative return during the learning process, by randomly extracting data from the experience buffer pool for repeated training and learning. Finally, unmanned ships can avoid obstacles and reach the destinations with the help of the Ship Action Controller.

• AC Algorithm

AC Algorithm includes Actor network and Critic network, which respectively include online network and target network. The two networks have the same network structure and they adopt the experience replay technology to randomly extract ship sample data from the experience buffer pool for network training.

• Environment

The training environment of autonomous path playing model of unmanned ships mainly includes Ship Action Controller and Ship Navigation Information Fusion Module. When the ship performs the action the environment feedbacks the reward and the state of the ship.

• Ship Action Controller

The Ship Action Controller converts the model output action to deflection heading and speed increment. These actions indicate that the unmanned ship should perform in the current state.

• Ship navigation information fusion module

The module can receive and process global positioning system (GPS), automatic identification system (AIS), depth sounder, and anemometer in real time (the information can be integrated and displayed on the electronic chart platform), and provide information of the unmanned ships state, including the ship’s position, heading, speed, the distance between the ship and the obstacle, and the angle of the ship and the target point. 

• Experience replay memory

The function of this module is to store the state of the ship, the action, the reward, and the state of the next moment. When the maximum capacity of the experience buffer pool is reached, the old data will be replaced by the new data, and the buffer pool is updated in this way.

The DDPG algorithm pseudo code is as follows.
**Algorithm 1: Pseudo code of the DDPG algorithm.**1: Randomly initialize Critic network $Q(s,a|\theta^{Q})$ and Actor network $\mu (s|\theta^{\mu })$ with weight parameters $\theta^{Q}$ and $\theta^{\mu }$. 2: Initialize target network Actor $Q^{\prime }(s,a|\theta^{Q^{\prime }})$ and Critic $\mu^{\prime }(s|\theta^{\mu^{\prime }})$ with weight parameters $\theta^{Q^{\prime }}\leftarrow \theta^{Q}$ and $\theta^{\mu^{\prime }}\leftarrow \theta^{\mu }$. 3: Initialize experience replay memory D. 4: for t = 1 to M do 5: Initialize the random process ℕ in the action exploration strategy. 6: Input initial unmanned ships and environment observation state $s_{1}$: ship latitude and          Longitude, ship heading, ship speed, angle with target point, distances from obstacles. 7: for t = 1 to T do 8:    Choose ship heading and ship speed $a_{t}=\mu (s_{t}|\theta^{\mu })+{\mathbb{N}}_{t}$ based on current strategy $\mu (s_{t})$
      and exploring noise ℕ. 9:    Implement output action $a_{t}$ to get the reward $r_{t}$ and the new state $s_{t+1}$. 10:   Save transition $\left(s_{t},a_{t},r_{t},s_{t+1}\right)$ into D. 11:   Sample random batch of N transitions $\left(s_{i},a_{i},r_{i},s_{i+1}\right)$ from D. 12:   Set $y_{i}=r_{i}+\gamma Q^{\prime }(s_{i+1},\mu^{\prime }(s_{i+1}|\theta^{\mu^{\prime }})|\theta^{Q^{\prime }})$ 13:   Update online Critic network by minimizing loss: $L=\frac{1}{N}{\sum_{i}\left(y_{i}-Q(s_{i},a_{i}|\theta^{Q})\right)}^{2}$ 14:     Update online Actor network using sampled policy gradient: $\nabla_{\theta^{\mu }}J\approx \frac{1}{N}\sum_{i}\nabla_{a}Q(s,a|\theta^{Q}){|}_{s=s_{i},a=\mu (s_{i})}\nabla_{\theta^{\mu }}\mu (s|\theta^{\mu }){|}_{s_{i}}$ 15:   Update target Actor network and target Critic network: $\begin{array}{l} \theta^{\mu^{\prime }}\leftarrow \tau \theta^{\mu }+(1-\tau )\theta^{\mu^{\prime }}\\ \theta^{Q^{\prime }}\leftarrow \tau \theta^{Q}+(1-\tau )\theta^{Q^{\prime }} \end{array}$ 16: end for17: end for

#### 3.3.2. Structural Design of AC Algorithms

**Figure 6:**
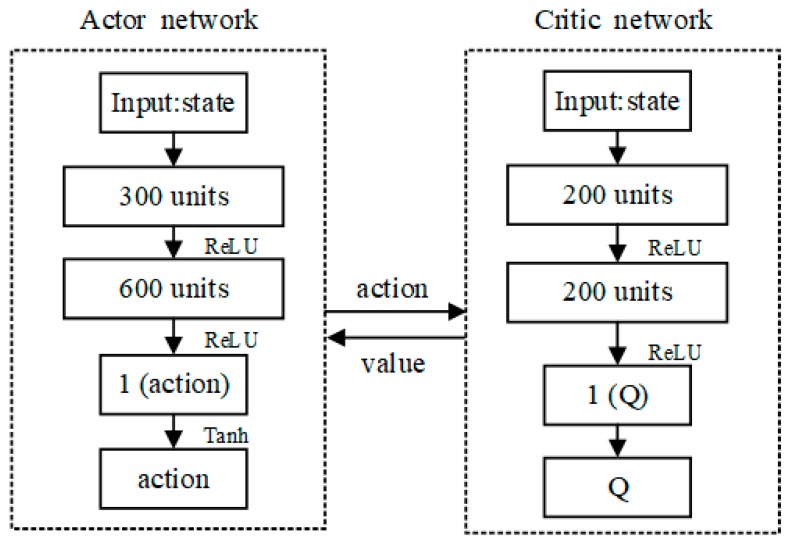
Network structure of AC algorithms.

The input parameters of the Actor network and the Critic network are all from the ship state data that were provided by the Ship Navigation Information Fusion Module. The Actor network and the Critic network continuously calculate and update their network parameters by continuously inputting ship state information, and finally get better network output results, which are respectively the action of unmanned ships and the value of action. Figure 6 shows the specific network structure of the AC algorithm.

The Actor network structure contains two fully connected hidden layers. The number of neurons is 300 and 600, respectively. The output nodes of each hidden layer are nonlinearly processed by the activation function to limit the number of output actions to one. First, the Actor network enters the initial ship state information $s_{t}$, calculates it through two hidden layers of neurons, and then uses the ReLU activation function to limit the output of each layer. At the same time, in the last layer of the network, the Tanh activation function is used to limit the network single output action value $a_{t}$ between [−1, 1], and the Ship Action Controller is then used to convert the network output action into the actual operation action.

The Critic network and The Actor network have the same network structure. The number of neurons of the two hidden layers is 200, and the output node of each hidden layer also adopts the activation function for non-linear processing. The evaluation value of the output action of Actor network is the final network result, which is known as the Q value. Different from the input parameters of the Actor network, the Critic network takes the initial state of the ships $s_{t}$, and the output action of the Actor network $a_{t}$ as the input parameters. After two hidden layers’ calculation, the output is processed by ReLU activation function, but when the final action value Q is output, no activation function is used to ensure that the output result of network is the definite action value. Additionally, the action value is used to evaluate the output action of Actor network.

#### 3.3.3. Action Strategy Design

In the process of DRL, the relationship between exploitation and exploration needs to be correctly handled. The appropriate action exploration strategy can enable the agent to try more new actions, so as to avoid falling into local optimization. In this paper, the action strategy of unmanned ships mainly includes action control strategies and action exploration strategies. When designing a neural network structure, the random noise is added into the output action of the Actor network as an action exploration strategy, and the output action is converted into specific execution as the control strategy according to the actual physical meaning.

• Action control strategy

In the aspect of action control strategy, one of the output actions of Actor network is used to indicate the deflection heading $a_{steering}$ and the Tanh activation function is used to control the range of the output value is [−1,1]. $D_{\max}$ indicates the maximum deflection angle, the value range is $\left[0^{{}^{\circ}},35^{{}^{\circ}}\right]$, and steering is used to represent the actual deviation sailing value of the ships. The calculation formula is as follows:

(2) $$steering=Tanh(a_{steering})D_{\max}$$

Another output action of the Actor network is ship speed increment $a_{shifting}$, which is processed by activation function Tanh. The output range is [−1,1]. $V_{\max}$ is the maximum value of the ship’s speed change, the range of value is [0,15] kn, and shifting is the actual change of ship speed. The calculation formula is as follows:

(3) $$shifting=Tanh(a_{shifting})V_{\max}$$

• Action exploration strategy

In the aspect of action exploration strategy, the effective implementation of action exploration in the continuous control space can enable the unmanned ships to find a better action since DDPG is an off-strategy algorithm. The method of adding random noise into the output action of the neural network is used to realize the exploratory process of the action. Random noise is defined, as follows:

(4) $$\mu^{\prime }(s_{t})=\mu (s_{t})+{\mathbb{N}}_{t}$$

where, $\mu^{\prime }$ is the exploration strategy and ${\mathbb{N}}_{t}$ is the added random noise.

The Ornstein–Uhlenbeck (OU) process is a sequential correlation process. It is often used as the random noise of DDPG algorithm and it has good effect in continuous action space. In this paper, this method is used as random noise of unmanned ships action strategy and it is specifically defined as:

(5) $$dx_{t}=\theta (\mu -x_{t})dt+\sigma dW_{t}$$

In the above formula, θ is the speed of the variable approaching the average value, μ is the action mean value, σ is the fluctuation degree of the random process, and $W_{t}$ is the Wiener process.

#### 3.3.4. Data Preparation

Based on the ship AIS system, this paper collects the real AIS data of 50 ships from August 1st to 15th, 2018 in the Dalian-Yantai route. Through the analysis of data, complete ship navigation data can be obtained, for example: Longitude, Latitude, speed over ground (SOG), course over ground (COG), heading (HDG), and other information. Table 1 shows the main information of AIS.

This paper mainly selects the longitude, latitude, heading, and speed information of the ship as the input parameters of the model. The trajectory of each ship can be clearly displayed since the data is collected in real time. Their total mileage is 133,550 nautical miles and the data volume is around 3G. This paper randomly selects the data of 20 ships from the first 50 ships as the input data of the model training since these data are collected in real time. These data were normalized prior to model training to speed up the training of the model and improve the accuracy of the model.

#### 3.3.5. Design of Reward Function

On the one hand, the unmanned ships needs to maintain the exploration ability to obtain better action, on the other hand, it also needs to use the learned action to obtain more reward from the environment, in order to fully explore the environment space to obtain the optimal action strategy.

The reward function is also known as the immediate reward or enhancement signal R. When an unmanned ship performs an action, the environment will make feedback information based on the action to evaluate the performance of the action. The environment and decision makers design the reward function. It is usually a scalar, with positive value as reward and negative value as punishment. It is very important to obey the navigation rules for the practicability of the algorithm in the autonomous path planning process of unmanned ships. 

**Figure 7:**
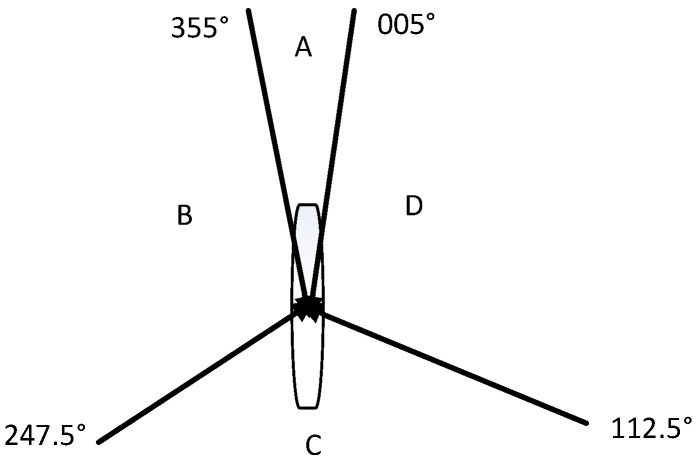
Ship encounter situation chart.

According to the COLREGS, the ship encounter situation can generally be divided into three types: head-on situation, crossing situation, and overtaking situation. Figure 7 shows the situation of the ship encounter situation.

Different ship encounters will be divided according to COLREGS when the sea environment has good visibility, as shown in Figure 7. The range of the A region is (0°, 005°) and (355°, 360°); it is called head-on situation. The ship should take a steering action that is greater than 15° to the right. The B region is a crossing situation with a range of (247.5°, 355°). The C region is the overtaking situation, and the range is (112.5°, 247.5°). The ship is overtaken and it does not usually take action. The D region is a crossing situation with a range of (005°, 112.5°). The ship should take right-turning action. In addition, in situation where the visibility of the sea environment is restricted, there will be no responsibility separation between the stand-on vessel and the give-way vessel. The COLREGS made the following rules for the situation of the ship at this time: For coming ships in the range of (0°, 90 °) and (270°, 360°), the self-ship will turn to the right. Self-ship takes a turn towards other ships for coming ships in the range of (90°, 180°) and (180°, 270°). We mainly study the situation of good visibility at sea in this article.

The rules need to be mathematically quantified and the rules are converted into navigational restrictions in order to integrate the COLREGS and crew’s experience into the model. Set the navigation limit area when the distance between the ship and the other ship is less than six nautical miles, otherwise it will not be set. The following describes the rule conversion in the three scenarios:

1. Head-on situation. Under this circumstance, both ships should take the action of turning to the right to avoid the boat. The method of rule conversion is as follows: a navigational limit line with a length of three times the ship length and a direction of 10° from the bow is drawn clockwise in the bow direction of the two ships. The environment will punish the ship if the ship crosses the navigation limit line. The specific performance is that the ship receives a negative reward.
2. Crossing situation. If there is a ship on the right side of the other ship, the ship should pass through the tail of the other ship. The method of rule conversion is as follows: draw a navigation limit line that is equal to four times the length of the ship in the direction of the bow of the other ship, to avoid the self-ship passing through the bow of the other ship. The ship will be punished and receive a negative reward if the ship crosses the navigation limit of the other ship.
3. Overtaking situation. The ship should pass from both sides of the other ship when the ship is behind the other ship and needs to overtake the other ship. The method of rule conversion is as follows: a navigation limit line of 1.5 times the length of the ship is drawn at the tail of the other ship in the vertical direction of the course. If the ship crosses the navigation limit line, it will be punished and get a negative return value.

For the conversion of crew’s experience, this paper adds a virtual area for obstacles based on crew’s experience in order to enable the ship to take actions to avoid obstacles in advance. When the distance between the ship and the obstacle is more than 1.5 times the length of the ship, the obstacle area is set, otherwise it is not set. The same as embedding COLREGS, we also convert the crew’s experience into a restricted area. We set the obstacle as an approximate regular pattern in order to simplify the calculation and unify the criteria (a circular). The method of setting the virtual obstacle area is as follows: the center of the obstacle is the circle and the longer side of the obstacle is used as the radius. Once the ship enters the obstacle area, the environment will punish it. In this case, the reward obtained by the ship is the inverse of the distance between the ship and the obstacle. The punishment is stronger when the ship is closer to the obstacle and, on the contrary, it will be smaller. The reward dynamically changes until the ship is outside the obstacle area.

By setting the navigation restriction area, the ship’s requirements for driving according to COLREGS and crew’s experiences are realized, thereby constraining and guiding the ship to select correct and reasonable behavior. The navigation rules are converted into navigational restrictions through the quantitative treatment of COLREGS and crew’s experiences. When the ship crosses a restricted navigation area or collides with an obstacle, it will be punished with a negative value. When the ship reaches the target point, the return value is positive. At the same time, the goal-based attraction strategy is adopted, and the reward is positively correlated with the distance between the ship and the target at the adjacent time. The reward function is designed, as follows:

(6) $$R_{t}\left\{ \begin{array}{l} 2,\;\;\;\;\;\;d_{t-goal}<D_{g-\min}\\ -1,\;\;\;\;\;\;d_{t-obs}<D_{o-\min}\\ (d_{t^{\prime }-goal}-d_{t-goal})-0.1,\;other \end{array}\right.$$

In the above formula, $d_{t-goal}$ is the distance between the ship and the target point at the current time t, the $d_{t^{\prime }-goal}$ is the distance between the ship and the target point at the previous time $t^{\prime }$, the $d_{t-obs}$ is the dangerous distance between the ship and the obstacle at the current time t, the $D_{g-\min}$ is the minimum threshold for the ship to reach the target point, and the $D_{o-\min}$ is the minimum dangerous distance threshold between the ship and the obstacle.

During the training process, when the ship reaches the target range, that is $d_{t-goal}<D_{g-\min}$, the reward is set to 2. When reaching the obstacle range, that is $d_{t-obs}<D_{o-\min}$, the reward is set to −1. In other cases, the attraction strategy is to attract the ship to the target point as soon as possible, in order to maximize the return value of the action. The current ship’s distance from the target point is subtracted from the distance between the ship and the target point at the previous time to determine whether the ship is approaching the target point. As the simulation environment is based on a 800 × 600 pixels two-dimensional map, 1 pixel represents 10m in the actual environment. Here, $\left(d_{t^{\prime }-goal}-d_{t-goal}\right)$ is normalized, that is, the result of dividing the distance between the current position of the ship and the target point by the distance of the diagonal of the two-dimensional map is used as the reward value, and the range of the reward value is between [0, 1]. At the same time, the reduction of 0.1 in each step is to reduce the number of steps to reach the target point and avoid the redundancy in the planned path. The reward is calculated as $(d_{t^{\prime }-goal}-d_{t-goal})-0.1$. There are many training rounds in the experiment. The current round ends when the ship reaches the target point. If it collides with obstacles, the ship is stepped back and re-selected action. At the same time, the maximum number of steps in each round is set to 400 steps. The current round is ended and the next round is re-entered when the maximum number of steps is reached.

### 3.4. Model Execution Process

**Figure 8:**
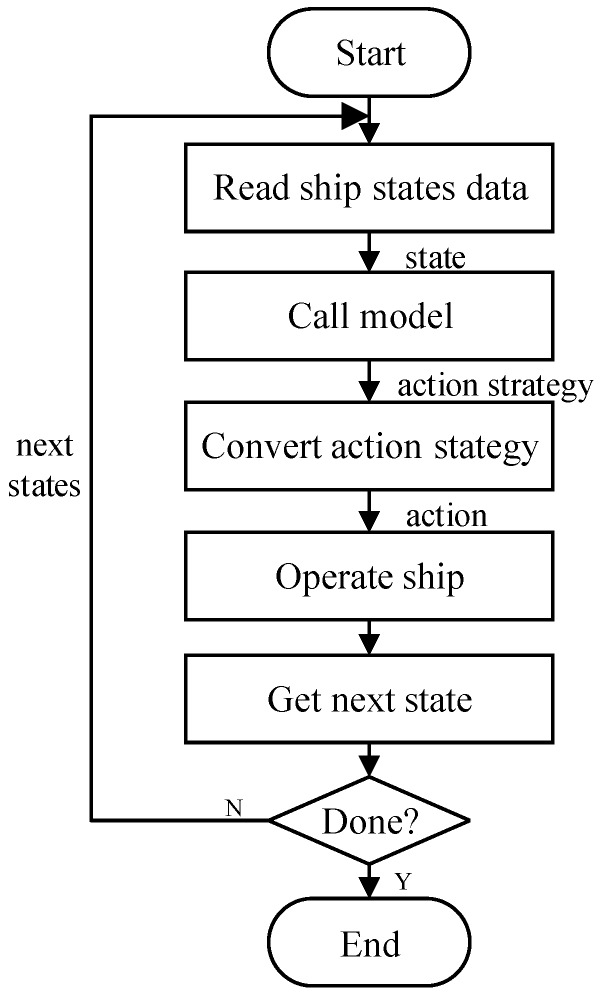
Execution process of unmanned ships path planning model.

The path planning model of unmanned ships based on the DDPG algorithm is used to abstract the real complex environment and then transform it into a simple virtual environment through the model. At the same time, the action strategy of this model is applied to the environment of electronic chart platform, so as to obtain the optimal planning track of unmanned ships and realize the learning process of end-to-end algorithm in the real environment. Figure 8 shows the execution flow of the autonomous path planning model for unmanned ships that are based on DDPG algorithm.

The execution process of the model for unmanned ships is described, as follows:

1. When the unmanned ships path planning program starts, the system reads the ship data through the ship navigation information fusion module.
2. The system invokes the model and takes the ship data as the input state and obtains the ship’s action strategy under the current state after the model processing and calculation.
3. According to the actual motion of the ship, the model further converts the action strategy into the actual action that the unmanned ships should take.
4. The ship action controller analyzes the acquired execution action and performs the action in the current unmanned ships state.
5. The model obtains the states information of the unmanned ships at the next moment after the execution action, and determines whether the ship state after the execution action is the end state.
6. The model will continue to use the state information of the ship at the next moment if it is not the end state, and then calculate and judge what action the ship should take at the next moment and cycle through it. If it is the end state, it indicates that the unmanned ships have completed the path planning task, and the model ends the calculation and the invocation.

In this section, first, the unmanned ship uses the obtained state information as the input to the algorithm. Second, the action strategy is obtained through training based on the navigation restricted and the reward function. Subsequently, the parameters of the model are updated with historical experience data until the cumulative return is at the maximum. Finally, the ship executes the action and changes its state, and it determines whether to end the execution process by judging the current state.

## 4. Simulation Results and Experimental Comparison

### 4.1. Definition of Model Input and Output Parameters

The input parameters of the model are ship data obtained from ship navigation information fusion module, which mainly includes ship’s own state, target point information, and surrounding information. The output of the model is the execution action of unmanned ship, including the deflection heading and the speed increment. Table 2 shows the input parameters names and definitions used in the model.

Here, the Angle between the current heading of the ship and the target point is designed as the input parameter, so that the unmanned ships can be quickly driven toward the target point, and the model training period is prevented from being too long. At the same time, the obstacle risk in the range of 1000 m is calculated and then stored in the set Tracks as the decision-making basis for ship obstacle avoidance. Among them, the set of distances from obstacles is differentiated according to the training situation. In the case where the self-ship heading extension line intersects with the other ship heading extension line, that is, on the premise that there is a danger of collision. If it is a single ship encounter, Tracks has only one distance information of other ships. Tracks contains the distance information of the most dangerous other ship if it is a situation where multiple ships meet. Here, the method of selecting the most dangerous other ship is: When the heading extension line of multiple other ships intersects the heading extension line of self-ship, we choose to store the smallest distance between the self-ship and other ship in Tracks. Finally, the above input states are respectively normalized to limit their range to [0, 1], thereby improving the calculation efficiency of the model. The deflection heading Steering and speed increment Shifting are used as the output action of the model, which realizes the direct conversion from the input state to the output action. Table 3 shows the output parameters definition.

According to the actual navigation situation, the maximum deflection heading of the unmanned ship is set to 35°. There is a situation that the ship might not be able to complete the currently required deflection angle but receive a new steering action due to the slower steering of the ship. Therefore, the same action accumulation method is used to calculate the heading deflection angle $d_{\psi }$. The heading deflection angle of the unmanned ship is obtained by Formula (7).

(7) $$d_{\psi }=\sum_{i=t}^{t+k-1}a_{i}$$

where, $a_{i}$ represents the unmanned ship deflection angle generated by the model at time i, and k represents the same action performed k(k>0) times in succession. 

In addition, the current ship’s heading $\psi_{d}$ can be obtained by summing the ship′s heading and deflection angle at the previous moment since the ship’s heading at the previous moment $\psi_{p}$ is known. The current heading of the unmanned ship is obtained by Formula (8).

(8) $$\psi_{d}=\psi_{p}+d_{\psi }$$

### 4.2. Model Training Process

Python language and electronic chart platform are used for model training and simulation experiments in order to verify the validity and feasibility of the unmanned ship autonomous path planning model based on DDPG algorithm. The deep learning framework TensorFlow trains the model. The model is designed using the python language, and the convergence and accuracy of the model training can be directly observed. The simulation environment is an electronic chart platform designed and developed based on Visual Studio 2013. Additionally, the state size of the simulation environment is a two-dimensional map of 800 × 600 pixels, and the range of motion of the ship is set to the size of the two-dimensional map. The ship is considered to have collided if the ship crosses the boundary of the two-dimensional map. This paper first uses the DDPG algorithm to train a global route from the start point to the end point, and then trains on the situation of encountering dynamic obstacles (other ships) during the voyage. We will mainly study the part of the ship that avoids obstacles and reaches the end point since the planning of the global static route is not the focus of this paper.

After many experiments, the better structure and parameters of the neural network are designed. The network structure of the Actor and the Critic both use a fully connected neural network with two layers of hidden layers. The Actor network hyper parameters are set, as follows: the numbers of neurons in the hidden layer are 300 and 600, respectively, the learning rate of the network is $10^{-4}$, the action of the output layer is the heading deflection and the speed increment respectively, and different activations are selected according to the difference of the output action range. The heading deflection uses the tanh activation function and its output range is [−1,1]; the speed increment uses the sigmoid activation function, and its output range is [0,1]. The Critic network hyper parameters are set, as follows: the number of neurons in the hidden layer is 200, the learning rate is $10^{-3}$, and the discount factor γ of the reward function is set to 0.9. In addition, the experience buffer pool size is set to 5000 and the batch learning sample size is 64. The random noise uses the method in Equation (5); the value of μ is set to 0, θ is set to 0.6, and σ is set to 0.3. The rate of the soft update method is τ=0.01. The Actor network and the Critic network both use the Adam network optimizer. To prevent infinite training, set the maximum number of steps per round to 400, for a total of 300 rounds. The parameters of the Actor network and the Critic network are updated every 1000 steps in order to further improve the accuracy of the model in the training process. Based on the above training conditions and parameters, this paper studies and trains the path planning model of the unmanned ship. The training process and results are described, as follows.

**Figure 9:**
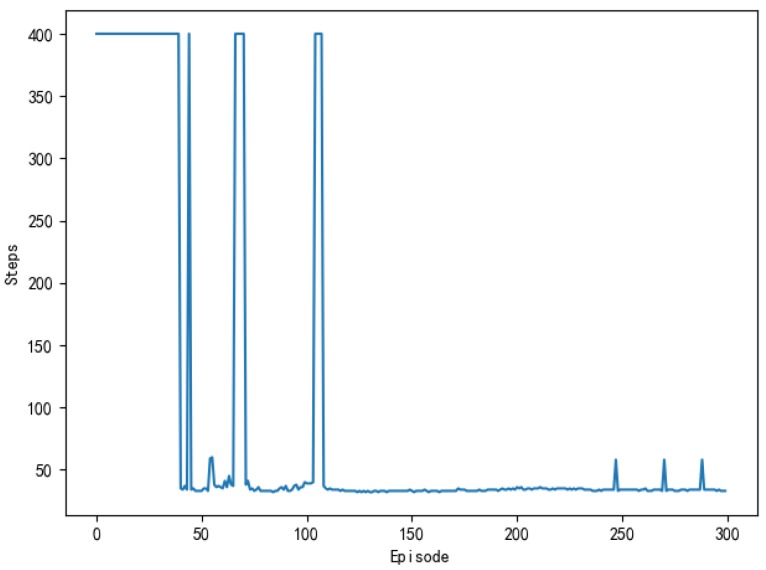
Number of steps per turn.

Figure 9 shows the number of training steps per round during the unmanned ships autonomous path planning process. It can be seen that, in about the first 40 rounds, the training steps of the model reach the maximum, which indicates that the model triggers the termination condition of training and it does not realize the path planning or falls into the local obstacle area. Between 45th and 110th round, the training steps of each round began to decrease, and the average training steps are maintained at 50 steps, which indicated that unmanned ships learn more and more action strategies and plan a complete path independently. After about the 115th round, the training steps of the model are kept below 40 steps in each round, which indicates that the unmanned ship has fully learned the optimal action strategy and it has no collision risk. In the vicinity of the 275th round, the number of training steps increased due to the exploratory strategy of the algorithm, which makes unmanned ships attempt random action.

**Figure 10:**
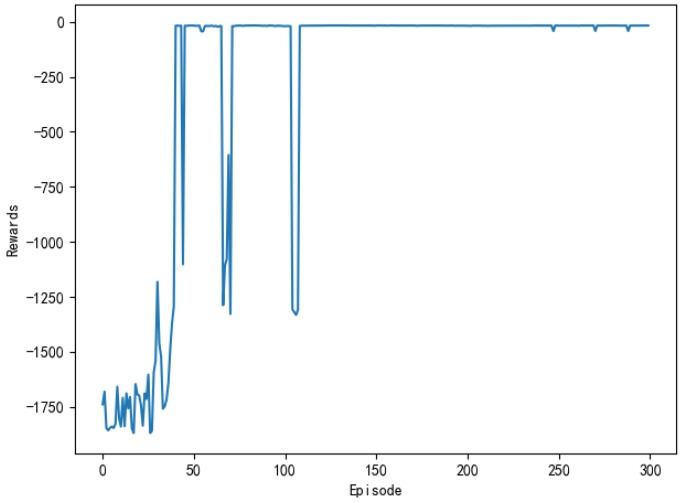
Cumulative reward per turn.

The purpose of the model is to improve the reward on the action strategy through continuous interaction with the environment. The greater the cumulative reward per round, the better the learning effect. Figure 10 shows the cumulative reward of each round of the model. In the first 40 rounds, the reward of the model is lower per round, and the fluctuation state is processed, which indicated that the unmanned ships has not found the correct path and is constantly trying new action strategy. Around the 45th round, the reward of the model began to increase, which indicated that a path to the target point is found. After the 115th round, the cumulative reward of the model per round is basically maintained at the maximum, indicating that the unmanned ships have found the optimal action strategy. The trend of cumulative reward per round is consistent with the change of steps per round when compared with Figure 9.

**Figure 11:**
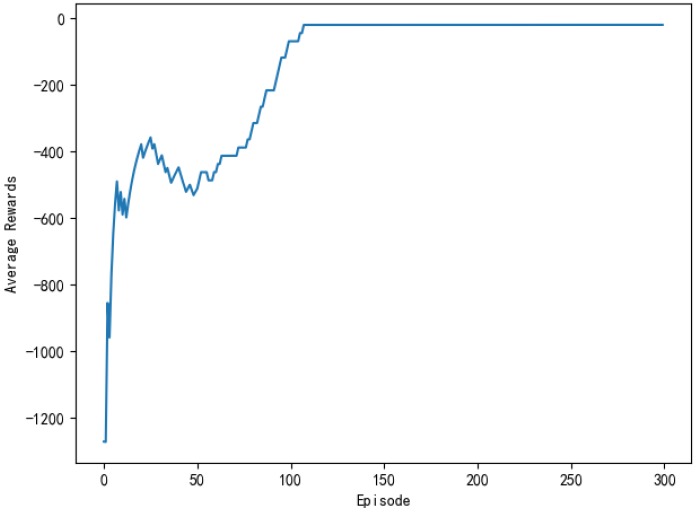
Average reward per rounds.

The average reward reflects the effect of the learning process and it also more directly observes the degree of change in the reward. Figure 11 shows the average reward of the model every 50 rounds. As can be seen from the figure, the general trend of average compensation is upward. About the 45th round, the growth rate of average rewards began to slow down and then gradually leveled off. After the 110th round, the average reward stabilized and then remained at a large value, which indicated that the model has found the optimal action strategy at this time.

### 4.3. Model Integration and Simulation Experiment

The model is tested and observed in the simulation environment in order to verify the validity and correctness of the autonomous path planning model for unmanned ships in Section 4.2. Firstly, the electronic chart platform and ship navigation rules are briefly introduced. Secondly, the model is validated in three different situations to observe whether the unmanned ship is running correctly according to the navigation rules. Finally, the other two unmanned ship path planning methods are selected as comparative experiments, and the training process and simulation results of the three methods are compared and analyzed.

#### 4.3.1. Verification Environment

It is difficult for traditional numerical simulation methods to accurately describe the environmental information because the unmanned ships have a wide range of work and its environment is complex. The electronic chart [37] platform is an important navigation tool for ships and other marine vehicles. It can provide real and complete environmental information needed in navigation, including land, ocean, water depth, obstacles, and islands, etc.

**Figure 12:**
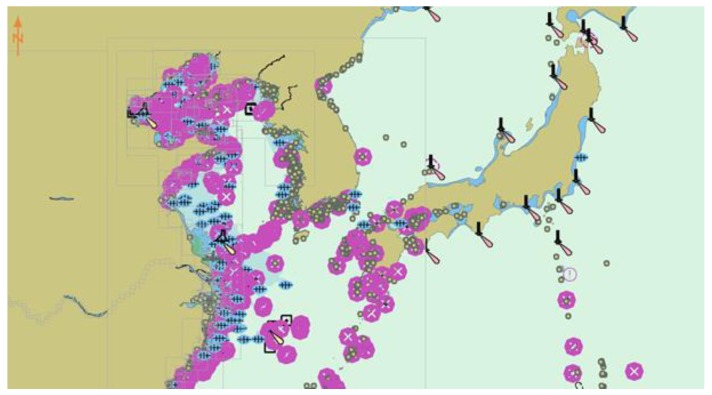
Electronic chart platform.

In this paper, the electronic chart platform that was developed in C++ language is used as the verification environment of the model, as shown in Figure 12. The platform has the following main features: (1) Displaying standard chart information, which can be used in any scale chart interface. (2) Use Microsoft Foundation Classes (MFC) as a dynamic link library, it provides a flexible and convenient interface. (3) Set the motion information of the ship and other ships, including longitude, latitude, heading, and speed. (4) Dynamic display of the track of all ships.

#### 4.3.2. Validation Results

The autonomous path planning model of unmanned ships is obtained through training, and the model is invoked by the electronic chart platform to further verify the effectiveness of the algorithm. In the electronic chart platform, the self-ship is indicated by black concentric circles and Ship-1, other ships are represented by triangles and Ship-i (i = 2, 3, 4, …), the yellow circle indicates the target point of the self-ship, and the purple circle indicates the target point of the other ship, static obstacles are indicated by irregular figures, and the trajectory of the ships are drawn during the movement. The motion parameters of self-ship are defined, as follows: the ship′s captain is set L=12.5 m, the ship’s width is set W=2.1 m, the heading change amount is $\psi_{m}=[-35^{{}^{\circ}},35^{{}^{\circ}}]$, and the shipping speed change amount is $v_{m}=\left[-15,15\right]\;\mathrm{kn}$. All other ships adopt uniform motion parameters. The length of the ship is $L_{o}=10.6\;m$, and the ship’s width is set $W_{o}=1.8\;m$. After setting the ship’s motion parameters, they travel to the set point according to the uniform linear motion.

The head-on situation, crossing situation, overtaking situation, and multi-ship encounter situation in the ship navigation process are tested, respectively, in order to verify whether the action strategy made by the model in this paper complies with the COLREGS. Here, the parameter information in the experiment process is uniformly introduced. Ship-1 represents the self-ship and Ship-i (i = 1, 2, 3, …) represents the other ship. Heading angle represents the initial angle of the ship and Ship Speed indicates the initial speed. The starting point and target point points are expressed in latitude and longitude.

(1) Head-on case

**Figure 13:**
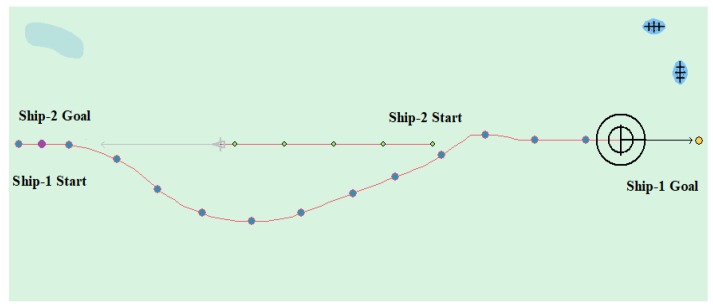
Verification result of ship trajectories in the head-on case.

Table 4 sets the motion parameters of the ships in detail. Figure 13 shows the experimental results. It can be seen from the waypoints information that the Ship-1 turned to the right according to the rules, successfully avoided the other ship, and then headed for the target position. In this case, the planned path length is 6.592 nautical miles.

(2) Crossing case

**Figure 14:**
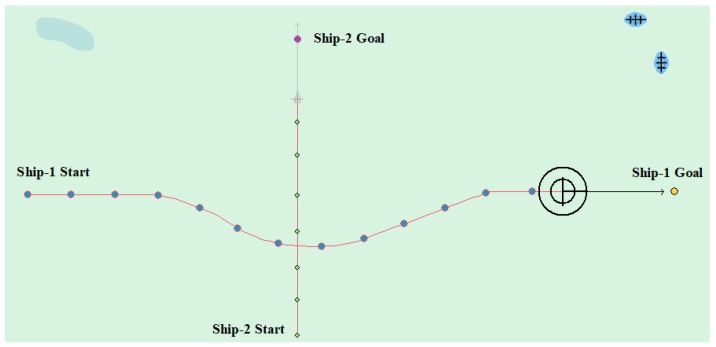
Verification result of ship trajectories in the crossing case 1.

**Figure 15:**
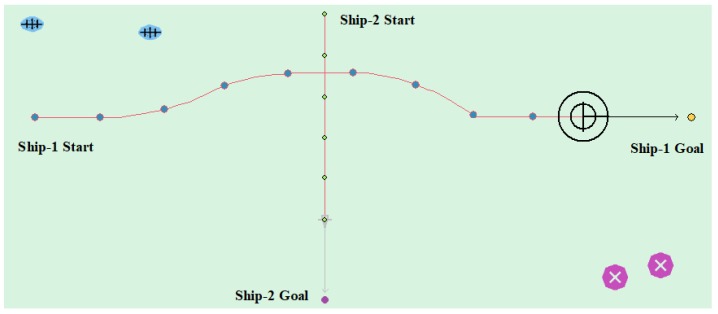
Verification result of ship trajectories in the crossing case 2.

Tested by the crossing of two ships, Table 5 and Table 6 show the specific ship motion parameters. Figure 14 and Figure 15 show the experimental results, respectively. In Figure 14, the other ship approached from the right side, where the Ship-1 acts as a give-way vessel with respect to the Ship-2, and then deflects the heading to the right according to rule and passes the stern of the Ship-2. In this case, the planned path length is 6.663 nautical miles. In Figure 15, the other ship approached from the left side, In this case, the planned path length is 6.462 nautical miles.

(3) Overtaking case

**Figure 16:**
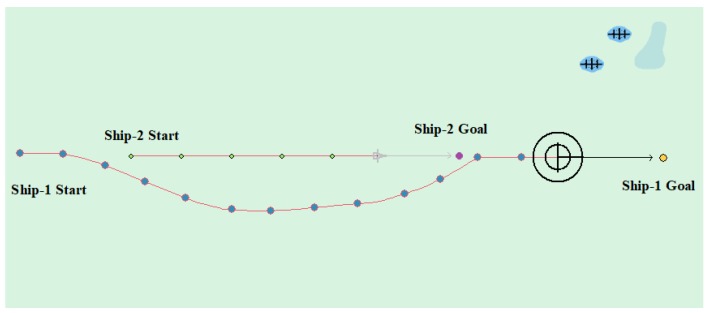
Verification result of ship trajectories in the overtaking case.

Table 7 shows the motion parameters of the two ships. From the test results and the waypoints information, Figure 16 shows that the Ship-1 is on the port side of the Ship-2, and the Ship-1 passes as the give-way vessel from the stern of the Ship-2. Ship-2 maintains heading as a stand-on vessel. In this case, the planned path length is 6.416 nautical miles.

(4) Multi-ship encounter case 1

**Figure 17:**
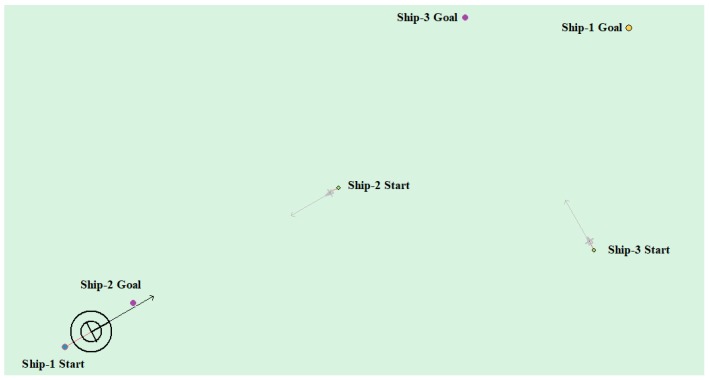
Multi-ship encounter case 1 scenario construction.

**Figure 18:**
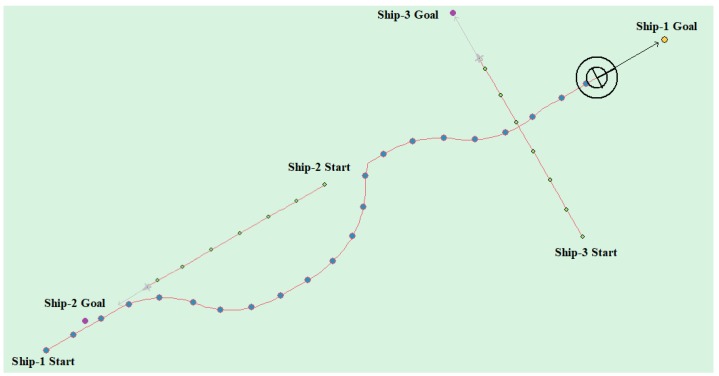
Verification result of ship trajectories in multi-ship encounter case 1.

Table 8 shows the motion parameters of the three ships. First, Ship-1 and Ship-2 form a head-on encounter, and then a crossing encounter with Ship-3. Ship-1 first takes the action of turning right, passing through the right side of Ship-2, then takes the action of turning right again, passing through the tail of Ship-3, and finally reaching the target point, according to the test results and waypoint information. Figure 17 shows the scene construction of multi ship encounter case 1, and Figure 18 shows the verification results of multi ship encounter case 1. In this case, the planned path length is 14.436 nautical miles.

(5) Multi-ship encounter case 2

**Figure 19:**
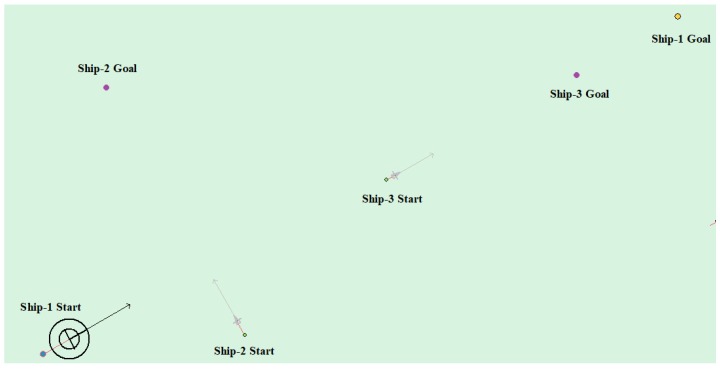
Multi-ship encounter case 2 scenario construction.

**Figure 20:**
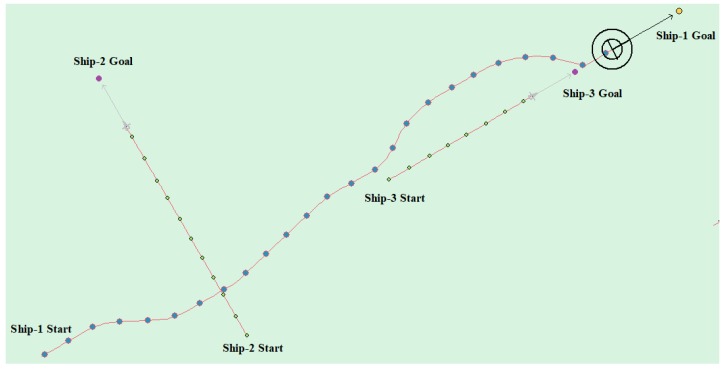
Verification result of ship trajectories in multi-ship encounter case 2.

Table 9 shows the motion parameters of the three ships. First, Ship-1 and Ship-2 form a crossing encounter situation and then form an overtaking situation with Ship-3. Ship-1 first takes the action of turning right, passing through the stern of Ship-2, then takes the action of turning left, passing through the right side of Ship-3, and finally reaches the target point, according to the test results and waypoint information. Figure 19 shows the scene construction of multi ship encounter case 2 and Figure 20 shows the verification results of multi ship encounter case 2. In this case, the planned path length is 13.651 nautical miles.

(6) Multi-ship encounter case 3

**Figure 21:**
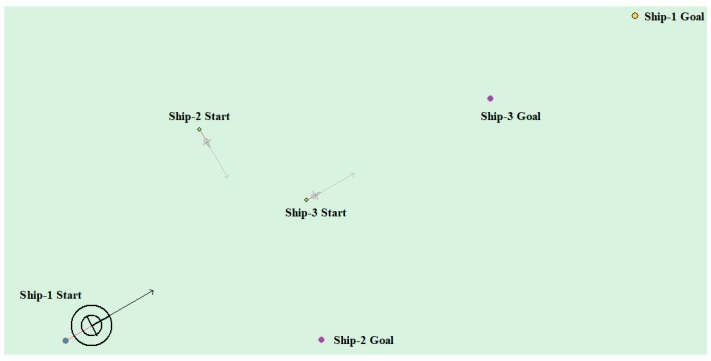
Multi-ship encounter case 3 scenario construction.

**Figure 22:**
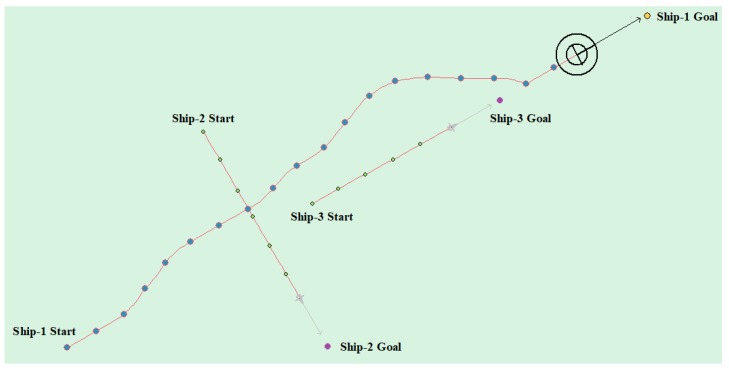
Verification result of ship trajectories in multi-ship encounter case 3.

Table 10 shows the motion parameters of the three ships. First, Ship-1 and Ship-2 form a crossing encounter situation and then form an overtaking situation with Ship-3. Ship-1 first takes the action of turning left, passing through the stern of Ship-2, then takes the action of turning left, passing through the right side of Ship-3, and finally reaches the target point, according to the test results and waypoint information. Figure 21 shows the scene construction of multi ship encounter case 2 and Figure 22 shows the verification results of multi ship encounter case 3. In this case, the planned path length is 15.264 nautical miles.

(7) Multi-ship encounter case 4

**Figure 23:**
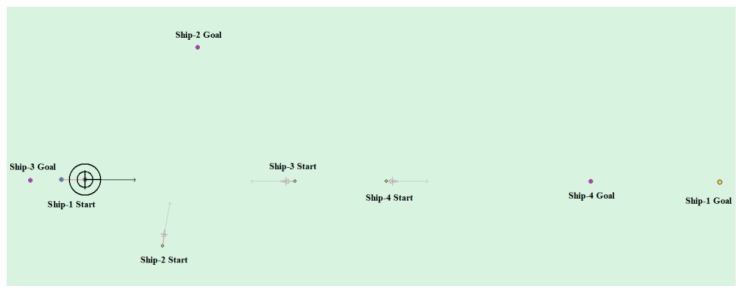
Multi-ship encounter case 4 scenario construction.

**Figure 24:**
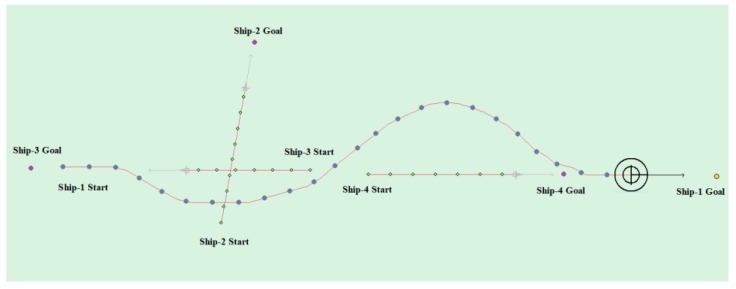
Verification result of ship trajectories in multi-ship encounter case 4.

Table 11 shows the motion parameters of the three ships. First, Ship-1 and Ship-2 form a crossing encounter situation, form a head-on encounter situation with Ship-3, and then finally form an overtaking encounter situation with Ship-4. According to the test results and waypoint information, Ship-1 first takes the action of turning right, passing from the stern of Ship-2 and the right side of Ship-3, then takes the action of turning left, passing from the left side of Ship-4, and then finally reaches the target point. Figure 23 shows the scene structure of case 3 of multi ship encounter and Figure 24 shows the verification results of case 4 of a multi ship encounter. In this case, the planned path length is 18.713 nautical miles.

Through the observation of the above verification test, it is shown that the unmanned ship based on the model that is proposed in this paper has better path planning effect in the case of single ship and multi-ship, and it can successfully avoid the obstacle according to the navigation rules. Finally, reach the target point. At the same time, by observing the time and path length used in path planning, it is further indicated that the path distance of the model planning is shorter and more in line with the actual navigation experience.

### 4.4. Improved Model of Autonomous Path Planning

The planned path is redundant and not sufficiently flat, although the unmanned ship autonomous path planning based on the DDPG algorithm is implemented in Section 4.3.2. By observing the training process in Section 4.2., it is found that the DDPG training time is longer, the convergence speed is slower, and the algorithm is easy to fall into the problem of local iteration. The above phenomenon is because the DDPG has no prior knowledge of the environment and the initial stage of learning can only randomly select actions. Therefore, the convergence speed of the algorithm is slow in complex environments. This section improves the DDPG and adds the APF method to obtain the unmanned ships path planning model based on APF-DDPG in order to improve the learning efficiency in the initial stage and speed up the convergence of the algorithm.

The APF is a method of virtual gravitational field and repulsive field, which has been widely used in real-time path planning of robots. The basic principle is to virtualize the simulation environment, and each state point in the environment has a corresponding potential energy value. The target point generates a gravitational potential field in the virtual environment, the obstacle generates a repulsive potential field in the virtual environment, and the total field strength is obtained by superimposing the gravitational field and the repulsive field. The object approaches the target point by using the gravitational field, and the repulsion field is used to avoid the obstacle. Generally, it is calculated by the following formula:

(9) $$U(s)=U_{a}(s)+U_{r}(s)$$

In the above formula, $U_{a}(s)$ is the potential energy value of the gravitational field at the point s, $U_{r}(s)$ is the potential energy value of the repulsive field at the point s, and U(s) is the potential energy of the point s. $U_{a}(s)$ and $U_{r}(s)$ are obtained by Formulas (10) and (11), respectively.

(10) $$U_{a}(s)=\frac{1}{2}k_{a}\rho_{g}^{2}(s)$$

In the Formula (10): $k_{a}$ is the scale factor of gravitational field and $\rho_{g}(s)$ is the minimum distance between the s and the target.

(11) $$U_{r}(s)=\left\{ \begin{array}{l} \frac{1}{2}k_{r}(\frac{1}{\rho_{ob}^{}(s)}-\frac{1}{\rho_{0}^{}}{)}^{2},\rho (s)<\rho_{0}\\ 0,\;\;\;\;\;\;\;\rho (s)\geq \rho_{0} \end{array}\right.$$

In the Formula (11): $k_{r}$ is the scale factor of repulsion field and $\rho_{ob}(s)$ is the minimum distance between the s and the obstacle, where $\rho_{ob}(s)$ is the obstacle influence coefficient. 

For the path planning problem of unmanned ships, the immediate reward r can only be obtained when the destination is reached or an obstacle is encountered. The sparsity of the reward function leads to low initial efficiency and multiple iterations. There are a large number of invalid iterative search spaces, especially for large-scale unknown training environments. Therefore, the APF is constructed according to the position information of the target point and the obstacle point. At this time, the potential field value of each state in the potential field represents the maximum cumulative return $V(s_{i})$ of the state $s_{i}$, and the relation expression is expressed as:

(12) $$V(s_{i})=|U(s_{i})|$$

In the Formula (12): $U(s_{i})$ is the potential field value of state $s_{i}$ in the virtual potential field environment. $V(s_{i})$ represents the maximum cumulative return when the optimal action is taken in state $s_{i}$.

The steps of the APF-DDPG based autonomous path planning method are as follows:

1. The potential field is constructed according to the target point and the obstacle in the virtual environment, and the gravity potential field with the target point as the center of the potential field is established.
2. Define the potential energy value $U(s_{i})$ in the potential energy field as the maximum cumulative report $V(s_{i})$ under state $s_{i}$, according to Equation (12).
3. The ship explores the environment from the starting point and selects the action in the current state $s_{i}$. The environment status is updated to state $s^{\prime }$ and an immediate return value is received r.
4. Update the Q value according to the state value function: $Q(s_{i},a)=r+\gamma V({s_{i}}^{\prime })$. Subsequently, update the online Critic network.
5. Observe whether the ship reaches the target point or reaches the set maximum number of learning. If the two meets one of them, the round of learning ends and the next iteration is started. Otherwise, return to step (3).

When the gravitational field is added to the target point position, the unmanned ship reaches the target point faster and the selection of the action strategy becomes more stable. The experimental procedure that is based on the APF-DDPG method is described below.

The path planning experimental parameters that are based on the APF-DDPG are the same as the DDPG parameters in Section 4.3.2. The APF parameters are set, as follows: $k_{a}=1.6$, $k_{r}=1.2$, $\rho_{0}=3.0$. We choose the environment of case (4), (5), and (7) in Section 4.3.2 as the comparison environment in order to better compare APF-DDPG with DDPG.

**Figure 25:**
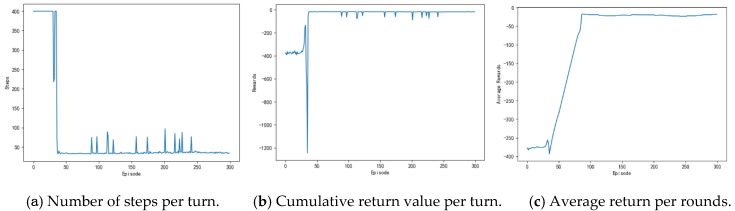
Artificial potential field-DDPG (APF-DDPG) training process.

Figure 25 shows the training process of APF-DDPG. From Figure 25a, it can be seen that the number of training steps in each round begins to decline and converge in the 43th round, and it fluctuates in the subsequent training process, which is caused by the action exploration strategy of the algorithm. Figure 25b shows the reward value of each round. The reward value of each round starts to increase and then reaches the maximum value from the training to the 43th round, indicating that a better action strategy is found at this time. Figure 25c shows the average reward value of each round, which rapidly increases and then stabilizes at the maximum value at the beginning of the 43th round.

**Figure 26:**
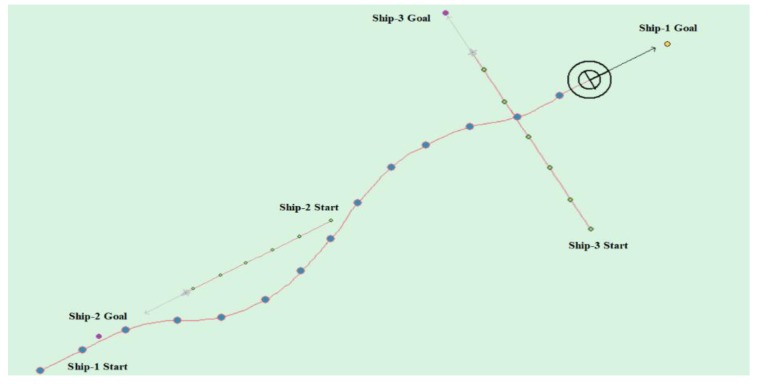
Experimental results based on APF-DDPG (a).

**Figure 27:**
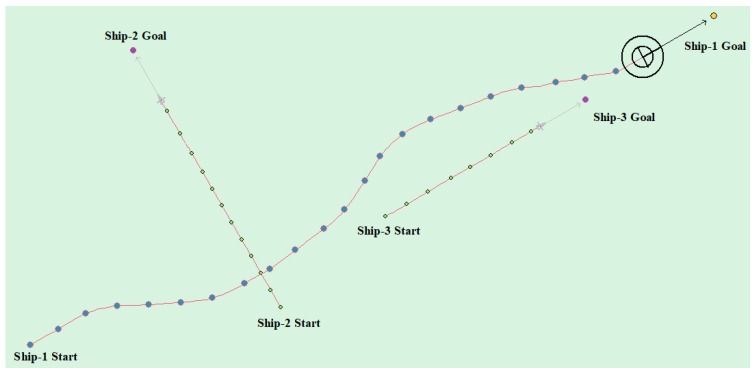
Experimental results based on APF-DDPG (b).

**Figure 28:**
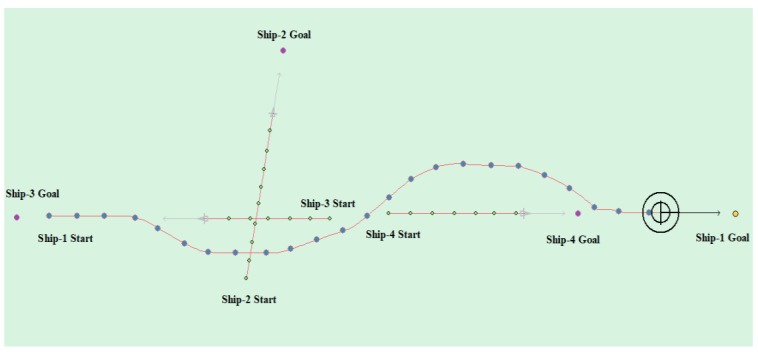
Experimental results based on APF-DDPG (c).

The result is shown in Figure 18 and Figure 26 as compared with the (4) experimental case in Section 4.3.2. Figure 18 has more redundant paths and it takes longer. The path in Figure 26 is smoother and the length of the planned path is shorter and takes less time. Similarly, when compared with the experimental case (5) (7) in Section 4.3.2, the results are shown in Figure 20, Figure 27, Figure 24 and Figure 28, respectively. It can be seen that APF-DDPG is better than DDPG in the distance and time of path planning. By comparing the DDPG and the APF-DDPG, it is shown that the APF-DDPG has better decision-making level and faster convergence speed.

### 4.5. Experimental Comparison and Analysis

Currently, there are five path planning methods comparisons and experimental analyses, as described in this section. These methods are called the DQN method, AC method, DDPG method, Q-learning method [18], and the path planning method based on APF-DDPG. The five methods are trained to obtain training steps, the reward, and the average reward in the case that the ship’s motion parameters and the surrounding environment are the same. Analyze and compare the experimental process and performance data of unmanned ships autonomous path planning during the training phase.

The five comparison methods set the same neural network structure and network parameters, the discount factor of the reward function is set to 0.9, and the experience buffer pool size is set to 5000. Among them, the Q-learning algorithm and DQN algorithm discretize the deflection heading action into 70 deflection angle values in the range of [−35°, 35°], and other algorithms directly output the specific action value.

**Figure 29:**
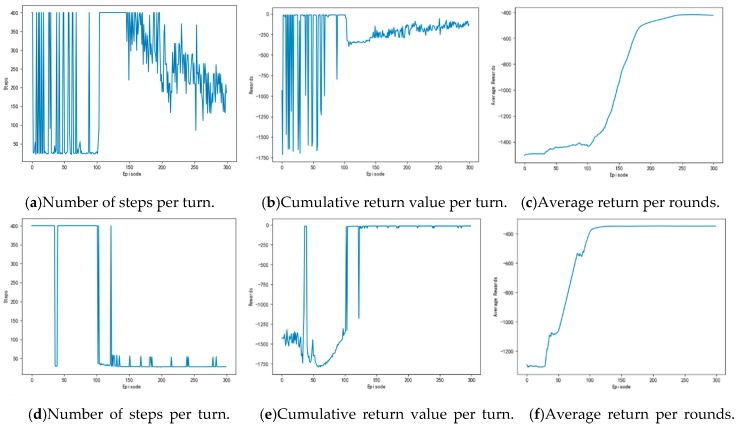

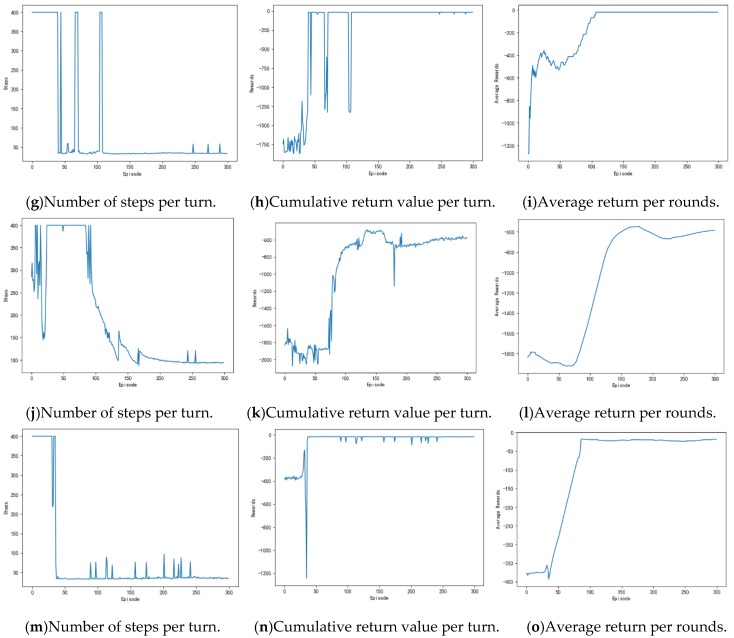
Comparative experiment of different path planning methods.

**Figure 30:**
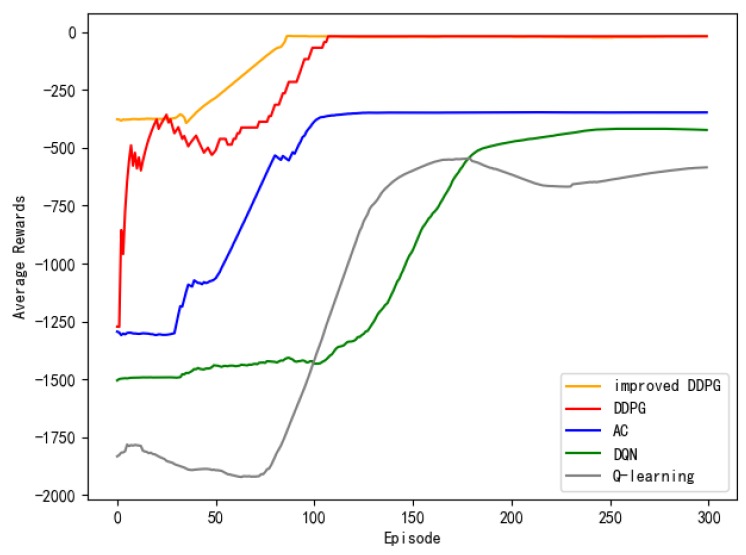
Comparison of average returns of five algorithms.

Figure 29 shows the contrast experiment of autonomous path planning for unmanned ships. Figure 29a–c are the results of an experimental of unmanned ships path planning based on DQN. Figure 29d–f are the results of an experimental based on AC. Figure 29g–i are based on the DDPG. Figure 29j–l are the results of an experiment based on the Q-learning. Figure 29m–o are based on the experimental results of the APF-DDPG. Figure 30 is a comparison of the average reward of the five methods.

(a), (d), (g), (j), and (m) in Figure 29 are the experimental results of the number of steps per round obtained while using the DQN, the AC, the DDPG, the Q-learning, and the APF-DDPG, respectively. By longitudinally comparing the number of execution steps per round, it can be found that the number of steps per turn based on the DQN (a) starts to decrease at about the 150th round, while the number of steps per turn based on the AC (d) and the DDPG (g) begins to decrease and then gradually converges around the 100th round. The number of steps per turn based on the DQN does not converge to the minimum number of steps, but the AC and the DDPG have both converged to the minimum number of steps, and the DDPG produces fewer fluctuations in the latter process. In addition, the number of steps per round based on the Q-learning (j) begins to decrease around the 150th round, and the number of steps per round based on the APF-DDPG (m) begins to decrease around the 43th round. By vertically comparing the five methods, it can be found that the path planning method that is based on the APF-DDPG reaches the target point with fewer rounds, and the convergence speed is faster, which is better than the other algorithms in terms of stability.

(b), (e), (h), (k), and (n) in Figure 29 are the experimental result of the reward per round obtained by the DQN, the AC, the DDPG, the Q-learning, and the APF-DDPG, respectively. The round reward based on the DQN (b) and the AC (e) starts to increase at about the 100th round, and then gradually reaches the maximum reward, and the round reward based on the DDPG (h) starts to increase at about the 50th round and then gradually stabilizes at the maximum reward, which indicated that the unmanned ships while using DDPG found the optimal action strategy faster. The round reward based on the DQN continuously fluctuates and does not reach the maximum reward, which indicates that the unmanned ships has not learned the optimal behavior strategy, and the AC has found the maximum reward value, indicating that the optimal behavior strategy has been learned. In addition, the round reward based on Q learning (k) has been low in the initial round, indicating that the correct action has not yet been learned and is in the exploratory stage. The 100th round of the award began to gradually increase, but ultimately did not reach the maximum reward. The round reward that was based on the APF-DDPG (n) began to increase at the 43th round and reached the maximum reward value faster. Longitudinal comparison of the five experimental processes shows that the APF-DDPG achieves the maximum round bonus value with the least amount of time and it is consistently maintained. At the same time, the APF-DDPG is more stable than the other algorithms in the later stage, which indicates that the unmanned ships has learned the optimal behavior strategy.

(c), (f), (i), (l), and (o) in Figure 29 is the experimental result of the average reward per round obtained by the DQN, the AC, DDPG, the Q-learning, and the APF-DDPG, respectively. By observing the average reward, it is more intuitive to judge the efficiency and accuracy of the convergence of the three algorithms. By observing (c), it can be known that the average reward that is based on the DQN starts to increase at about the 110th round and tends to be stable around the 180th round. By observing (f), the average reward that is based on the AC is the 40th round began to increase and then stabilized around 130th round. By observing (i), the average reward that is based on the DDPG of this paper started to increase at about the 10th round, and the downward trend appeared around the 50th round. This is because the random exploration strategy of the algorithm causes the reward of each round to fluctuate, but around the 115th round, the average reward reaches the maximum. In addition, the average reward based on Q learning (l) showed a downward trend at the beginning, which indicated that it was in the exploration stage. Subsequently, there was an increase in the 86th round, but did not reach the maximum average reward finally. By observing (o), the average reward that is based on the APF-DDPG increases at the 43th round and reaches the maximum average reward value faster, which converges faster than other algorithms. Longitudinal comparison of five experimental processes shows that the APF-DDPG achieves the maximum average reward with the least amount of time and it is consistently maintained.

Figure 30 is a comparison of the average reward for the five path planning methods. It can be seen from the figure that the Q-learning and DQN methods that are based on discrete motion space converge slowly, starting to gradually increase in the 80th to 100th rounds, and fail to reach the maximum draw value. Based on the AC and DDPG methods, convergence begins on the 10th to 50th rounds. The maximum average reward value is greater than the DQN and Q-learning methods, and the DDPG algorithm has reached the maximum average reward value. The average reward value of the APF-DDPG method is higher than the other methods at the beginning, which indicated that the initial convergence rate is improved after the artificial potential field method is added. At the same time, the APF-DDPG method reached the maximum average reward value in the 70th round. In summary, the APF-DDPG method is superior to other methods in terms of convergence speed and stability.

Experimental data from five methods were extracted, and the comparison is made from the total iteration time, the optimal decision time, convergence steps, and the number of obstacle collisions to further illustrate the accuracy and effectiveness of the improved model. As shown in Table 12, DQN and Q-learning based on discrete motion space have greater overhead in terms of training time and convergence speed. In contrast, AC and DDPG have better training time and convergence effects. When compared to the above method, the decision method using APF-DDPG has less iteration time and optimal decision time. In addition, the convergence speed of the method is improved, and the trial and error rate is reduced. The simulation results show that the APF-DDPG has higher convergence speed and stability.

## 5. Conclusions

For the traditional path planning algorithm, the historical experience data cannot be recycled and used for online training and learning, which results in low accuracy of the algorithm, and the actual path of the plan is not smooth and redundant. This paper proposes an unmanned ships autonomous path planning method based on the DDPG algorithm. First, the ship data are acquired based on the electronic chart platform. Secondly, the model is designed and trained in combination with the COLREGS and crew experience, and validated in three classical encounter situations on the electronic chart platform. The experiments show that unmanned ships take the best and reasonable action in unfamiliar environment, successfully complete the task of autonomous path planning, and realize the end-to-end learning method of unmanned ships. Finally, by combining APF and DDPG, an unmanned ship autonomous path planning method that is based on improved DRL is proposed. The improved DRL is compared with other classical DRL methods. The results show that the improved DRL has faster convergence speed and accuracy, can achieve continuous operation output, and has less navigation error, which further validates the effectiveness of the proposed method. However, the ship’s motion model and the actual verification environment are not considered in this paper. How to consider the motion model of a ship in a complex sea area and then verify it in a real environment is the focus of the next research in this paper.

## Figures and Tables

**Table 1:** Automatic identification system (AIS) data attribute table.

ID	Data Type	Data Value	Data Sources
1	Longitude	121°30.43′ E	AIS
2	Latitude	37°47.30′ N	AIS
3	SOG	17.5	AIS
4	COG	10.70°	AIS
5	HDG	10.00°	AIS
6	DRIFT	0.7°	AIS

**Table 2:** Input state definition.

Name	Parameters	Description
Longitude	[0°, 180°] E, [0°, 180°] W	Ship longitude
Latitude	[0°, 90°] N, [0°, 90°] S	Ship latitude
Direction	[0°, 360°]	Ship heading
Speed	[0, 30] kn	Ship speed
Angel	[0°, 360°]	Angle with target point
Tracks	(0, 1000)/m	Array of distances from obstacles

**Table 3:** Output state definition.

Name	Parameters	Description
Steering	[−1, 1]	Deflection angle (−1 for right full rudder, 1 for left full rudder)
Shifting	[−1, 1]	Speed increment (−1 for full deceleration, 1 for full acceleration)

**Table 4:** Setting of ship in head-on case.

	Starting Point	Target Point	Heading Angle	Ship Speed
Ship-1	(N 38.104, E 121.105)	(N 38.104, E 121.134)	90°	28 kn
Ship-2	(N 38.104, E 121.113)	(N 38.104, E 121.106)	270°	18 kn

**Table 5:** Setting of two ship in crossing case 1.

	Starting Point	Target Point	Heading Angle	Ship Speed
Ship-1	(N 38.104, E 121.105)	(N 38.104, E 121.134)	90°	28 kn
Ship-2	(N 38.193, E 121.112)	(N 38.221, E 121.112)	0°	18 kn

**Table 6:** Setting of two ship in crossing case 2.

	Starting Point	Target Point	Heading Angle	Ship Speed
Ship-1	(N 38.104, E121.105)	(N 38.104, E 121.134)	90°	28 kn
Ship-2	(N 38.221, E121.112)	(N 38.193, E 121.112)	180°	18 kn

**Table 7:** Setting of ship in overtaking case.

	Starting Point	Target Point	Heading Angle	Ship Speed
Ship-1	(N 38.203, E 121.134)	(N 38.203, E 121.246)	90°	28 kn
Ship-2	(N 38.203, E 121.137)	(N 38.203, E 121.236)	90°	18 kn

**Table 8:** Setting of ship in multi-ship encounter case 1.

	Starting Point	Target Point	Heading Angle	Ship Speed
Ship-1	(N 38.203, E 121.134)	(N 38.245, E 121.257)	60°	28 kn
Ship-2	(N 38.220, E 121.174)	(N 38.221, E 121.152)	240°	18 kn
Ship-3	(N 38.232, E 121.236)	(N 38.254, E 121.232)	330°	18 kn

**Table 9:** Setting of ship in multi ship encounter case 2.

	Starting Point	Target Point	Heading Angle	Ship Speed
Ship-1	(N 38.203, E 121.134)	(N 38.264, E 121.264)	60°	28 kn
Ship-2	(N 38.204, E 121.185)	(N 38.235, E 121.164)	330°	18 kn
Ship-3	(N 38.231, E 121.228)	(N 38.258, E 121.248)	60°	18 kn

**Table 10:** Setting of ship in multi ship encounter case 3.

	Starting Point	Target Point	Heading Angle	Ship Speed
Ship-1	(N 38.203, E 121.134)	(N 38.264, E 121.264)	60°	28 kn
Ship-2	(N 38.235, E 121.164)	(N 38.204, E 121.184)	150°	18 kn
Ship-3	(N 38.231, E 121.228)	(N 38.258, E 121.248)	60°	18 kn

**Table 11:** Setting of ship in multi ship encounter case 4.

	Starting Point	Target Point	Heading Angle	Ship Speed
Ship-1	(N 38.203, E 121.134)	(N 38.203, E 121.278)	90°	28 kn
Ship-2	(N 38.184, E 121. 138)	(N 38.246, E 121.141)	10°	18 kn
Ship-3	(N 38.203, E 121.164)	(N 38.203, E 121.131)	270°	18 kn
Ship-4	(N 38.202, E 121.245)	(N 38.201, E 121.257)	90°	18 kn

**Table 12:** Comparison of experimental data.

Comparative Experiment	Total Iteration Time (s)	Optimal Decision Time(s)	Convergence Steps	Number of Collisions
DQN	452.512	410.856	235	139
AC	361.219	332.463	136	84
DDPG	338.713	281.651	122	72
Q-learning	486.321	437.245	260	175
APF-DDPG	294.960	236.152	68	63
